# Perioperative Management of Presumed Schwartz–Jampel Syndrome During Complex Pediatric Spinal Fusion: A Case Report

**DOI:** 10.1155/cria/3939670

**Published:** 2026-09-06

**Authors:** Hannah Shreiner, Anna Booth, Amy McIntosh, Christopher McLeod

**Affiliations:** ^1^ Department of Orthopedics, Scottish Rite for Children, Dallas, Texas, USA; ^2^ Department of Orthopaedic Surgery, University of Texas Southwestern Medical Center, Dallas, Texas, USA, utsouthwestern.edu; ^3^ Department of Anesthesiology and Pain Management, University of Texas Southwestern Medical Center, Dallas, Texas, USA, utsouthwestern.edu

**Keywords:** case report, combined anterior and posterior spinal fusion, endotracheal tube obstruction, pleural adhesions, Schwartz–Jampel syndrome

## Abstract

Schwartz–Jampel syndrome (SJS) is associated with neuromuscular and structural abnormalities that can complicate perioperative and anesthetic management. The syndrome has been noted to create increased difficulty in anesthetic airway management with age. We report the perioperative course of a 13‐year‐old female with presumed SJS undergoing combined anterior and posterior spinal fusion for severe progressive congenital scoliosis. Preoperative evaluation revealed features predictive of a difficult airway, including limited mouth opening and restricted neck mobility, requiring video laryngoscopy for successful intubation. During the anterior thoracoscopic phase, dense pleural adhesions were encountered, requiring extensive pleural dissection and adhesiolysis to allow completion of the release of the anterior spinal column. After repositioning prone for the posterior procedure, the patient developed acute ventilation difficulty with markedly elevated airway pressures and severe hypercarbia. Flexible bronchoscopy revealed a nearly occlusive sanguineous mucus plug within the endotracheal tube, prompting abortion of the procedure. The patient returned 1 week later for completion of surgery, and difficulty with endotracheal tube depth was encountered due to a foreshortened trachea. She otherwise recovered without major complications. This case highlights the perioperative management of a complex pediatric neuromuscular scoliosis patient undergoing combined anterior and posterior spinal procedures. It underscores the importance of anticipating unexpected pulmonary complications in patients with difficult airway anatomy and severe restrictive lung disease. Heightened awareness of endotracheal tube obstruction and malposition is imperative.

## 1. Introduction

Schwartz–Jampel syndrome (SJS) is a rare autosomal‐recessive neuromuscular disorder characterized by short stature, a narrow mouth opening, limited range of motion due to myotonia, progressive joint contractures involving the hips, knees, and elbows that resemble pterygiums and, in some cases, spinal deformity [1, 2]. These clinical features can pose significant challenges for anesthetic airway management, as jaw muscle rigidity and restricted mouth opening may complicate tracheal intubation [3]. Notably, airway difficulty in patients with SJS appears to be progressive. Many patients undergo uneventful tracheal intubation earlier in life, with increasing difficulty developing as joint contractures and muscle rigidity become more pronounced with age [2, 4].

Beyond airway management, SJS presents additional anesthetic challenges related to musculoskeletal rigidity, spinal deformities, and impaired pulmonary mechanics, which may complicate patient positioning, ventilation, and surgical access during complex procedures [5, 6]. These considerations are particularly relevant in patients undergoing more complex orthopedic or thoracic surgical procedures, where restricted mobility and abnormal anatomy may increase perioperative risk. Despite these challenges, there is limited literature exploring intraoperative management of patients with SJS undergoing pediatric orthopedic surgery. We present a case of a pediatric patient with presumed SJS undergoing anterior spinal release and posterior spinal fusion. Though these pulmonary complications are not unique to SJS, this case highlights important anesthetic considerations beyond the airway and how common anesthetic issues can become exacerbated in the setting of complex positioning and surgical factors. The case submission adheres to CARE guidelines, and the CARE checklist is available as a supplemental document. Informed consent was obtained.

## 2. Case Presentation

**FIGURE 1:**
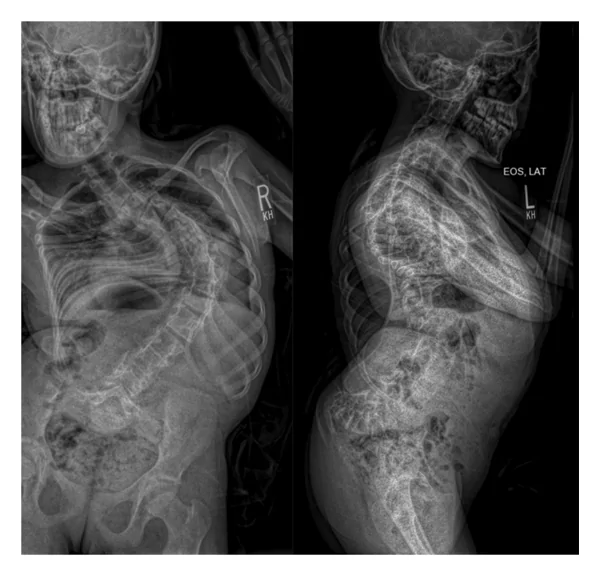
Preoperative radiographs, coronal and sagittal views showing significant thoracic spinal deformity.

A 13‐year‐old female, 134 cm, 25.6 kg, with a presumed diagnosis of SJS was admitted for video‐assisted thoracoscopic anterior spinal release and posterior spinal fusion (PSF) from T1 to L4 to correct severe progressive congenital scoliosis. She had been followed longitudinally at a single pediatric orthopedic facility since 5 months of age for congenital hand and foot deformities, joint contractures, and delayed motor milestones. She also had feeding difficulties at birth requiring gastrostomy‐tube placement at 6 months of age. Despite gross motor and cognitive delays noted early on in life, she currently has no cognitive impairment. A complete spine MRI and CT with 3D reconstructions were obtained at 11 years of age. They revealed a congenital cervical block vertebra at C2–C4 with fused spinous processes, cervical kyphosis at C4–C5, and thoracolumbar hyperlordosis from T1 to L4 due to multiple congenital abnormalities with incomplete segmentation of multiple vertebral segments and premature fusion of the posterior elements. This led to a severe progressive spinal deformity that required surgical intervention at 13 years of age (Figure 1).

The family met with three geneticists separately, and each proposed a syndromic diagnosis (Beals–Hecht syndrome, Pena–Shokeir syndrome, and SJS) when the patient was younger than 2 years of age. The family was recommended to pursue a comprehensive whole‐exome sequencing (WES) test to confirm SJS after the other syndromes were clinically excluded. WES was not pursued because of insurance‐related barriers, and the family also previously declined muscle biopsy for diagnostic confirmation. Over time, the most likely clinical diagnosis became SJS.

Prior to undergoing spinal surgery, the patient had numerous general anesthetics without any reported complications, including gastrostomy‐tube placement at 6 months of age, myringotomy with tube placement at 7 months, cleft palate repair at 2 years, full dental rehabilitations at 4 and 5 years, and femoral shortening osteotomy at 7 years. Each of these procedures was completed uneventfully. Her most recent airway instrumentation, with a supraglottic airway, occurred 5 years before spinal surgery.

Preoperative evaluation was significant for limited mouth opening, limited neck extension, and decreased thyromental distance. Despite limited mouth opening, she was a Mallampati I. Pulmonary function testing revealed severe restrictive lung disease with both FVC and FEV1 showing 22% of the predicted values for the patient’s age and height. The patient was premedicated with oral valium and underwent a preinduction intravenous catheter placement. She was monitored with standard ASA monitors and underwent induction with propofol, lidocaine, rocuronium, and fentanyl. She had easy mask ventilation; however, even with neuromuscular blockade, jaw tightness and a lack of tissue elasticity made instrumenting the airway challenging as mouth opening remained fixed. Placement of the Karl Storz C‐MAC D‐BLADE Pediatric was difficult and required horizontal rotation to position the blade into the oropharynx. A Cormack–Lehane classification Grade I view was obtained as an assistant placed significant cricoid pressure. A 5.5‐mm cuffed endotracheal tube was placed over a malleable stylet into the glottic opening and secured at 15 cm at the lips. A second peripheral intravenous catheter and left radial arterial line were placed following airway securement. Anesthesia was maintained with infusion of propofol, sufentanil, ketamine, and dexmedetomidine in the setting of intraoperative neurophysiological monitoring.

**FIGURE 2:**
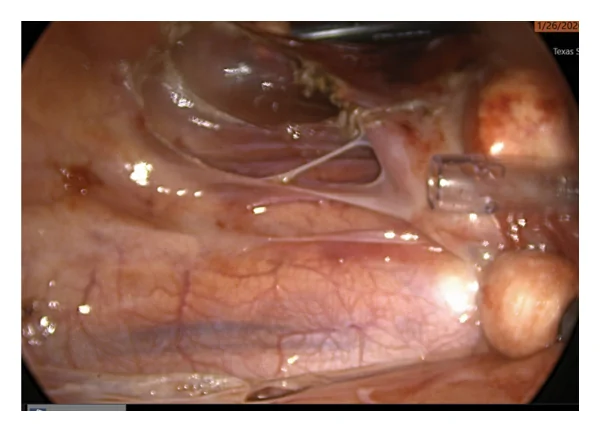
Intraoperative video‐assisted thoracoscopic lysis of lung adhesions.

The patient was positioned left lateral decubitus for thoracoscopic access. A two‐lung ventilation strategy was maintained, and adequate surgical exposure was obtained with a CO_2_ insufflation device, obviating the need for single lung ventilation. There were dense adhesions between the lung, chest wall, and diaphragm, requiring extensive adhesiolysis and surgical manipulation of the right lung before the thoracic spine could be adequately visualized for release of the anterior longitudinal ligament and partial thoracic discectomies (Figure 2).

**FIGURE 3:**
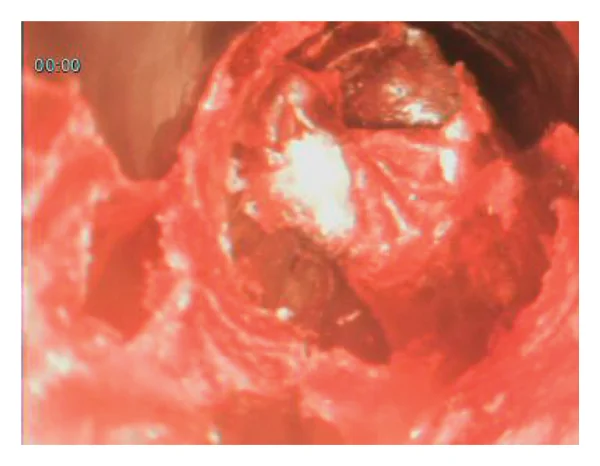
Nearly occlusive sanguineous endotracheal mucus plug noted with bronchoscopic evaluation.

**FIGURE 4:**
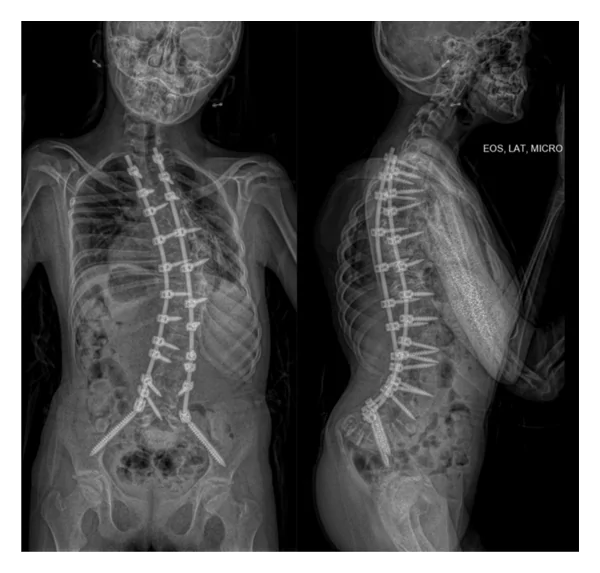
Postoperative radiographs, coronal and sagittal views showing correction in spinal deformity.

The patient was then positioned prone to undergo PSF surgery. Shortly after the posterior spine incision, high airway pressure was detected. The anesthetic agents were deepened, and a small dose of epinephrine was administered without resolution of ventilation difficulties. There was an inability to pass a soft suction catheter through the endotracheal tube, and flexible bronchoscopy revealed a nearly occlusive sanguinous mucus plug (Figure 3). The patient’s end‐tidal CO_2_ registered as high as 93 mmHg. The patient’s incision was urgently closed, and the patient was repositioned supine. Saline irrigation and suctioning of the endotracheal tube resolved the ventilation difficulties. Surgery was then aborted. Seven days later, the patient returned to the operating room for completion of the PSF from T1 to L4. Airway instrumentation was successful, but the patient was experiencing high airway pressures and elevated end‐tidal CO_2_ in the supine position. The patient demonstrated marked tracheal foreshortening and reduced glottic to carinal distance for a 13‐year‐old. A chest X‐ray was obtained, and the endotracheal tube was pulled back 2 cm, to a depth of 14 cm at the lips. With the endotracheal tube repositioned, the spinal fusion surgery was able to be successfully completed. The patient recovered uneventfully on the ward. Good correction was obtained in both coronal and sagittal planes, with instrumentation from T1 to L4 (Figure 4). Follow‐up visits have shown PFTs improved to FEV1 and FVC of 32% each. A chronological view of events is available in the patient timeline (Table 1).

**TABLE 1:** Patient timeline.

Time point	Key clinical events	Management and outcome
Infancy and early childhood	Feeding difficulties, cleft palate, congenital joint contractures, and delayed motor development	Gastrostomy placement, cleft‐palate repair, and ongoing orthopedic care
Ages 2–4 years	Evaluated by three geneticists for multiple congenital syndromes	Alternative diagnoses were clinically excluded. Schwartz–Jampel syndrome remained a presumptive diagnosis because confirmatory genetic testing was not completed
Age 11 years	Imaging demonstrated severe multilevel thoracolumbar deformity	Continued evaluation and planning for corrective spinal surgery
Age 13 years—preoperative evaluation	Progressive severe scoliosis with severe restrictive pulmonary disease	Thoracoscopic anterior spinal release and posterior spinal fusion were planned
Initial operation—anterior stage	Unexpected dense right‐sided pleural adhesions were encountered	Extensive adhesiolysis and anterior spinal release were performed
Initial operation—posterior stage	Severe ventilation difficulty developed after prone positioning. Bronchoscopy demonstrated a nearly occlusive sanguineous mucus plug	The patient was returned supine, the plug was removed, and ventilation improved. The posterior procedure was postponed
Seven days later—posterior fusion	Inadvertent mainstem intubation in the setting of a foreshortened trachea	The endotracheal tube was repositioned with improved ventilation, and posterior spinal fusion was completed successfully
Postoperative course and follow‐up	Uneventful recovery	Pulmonary function improved, with adequate correction of the spinal deformity

## 3. Discussion

SJS diagnosis is typically based on a clinical examination, supported by electromyography (EMG) demonstrating continuous or near‐continuous myotonic discharges, and molecular confirmation when available [4]. In genetically confirmed cases, pathogenic variants are typically identified in the heparan sulfate proteoglycan 2 (HSPG2) gene located on chromosome 1p36.1‐p35 [4, 7, 8]. The HSPG2 gene encodes perlecan, a key proteoglycan protein of basement membranes with roles in neuromuscular and skeletal development [9, 10]. Typical physical examination findings include myotonic facies, blepharophimosis, blepharospasm, micrognathia, limited mouth opening (trismus), short stature, muscle stiffness, and progressive joint contractures affecting the hips, knees, and elbows [2, 11, 12]. Many patients also demonstrate spinal deformity including rib anomalies, scoliosis, and kyphosis.

In our patient, the working diagnosis was clinical rather than genetically confirmed. EMG had also been performed earlier as part of an anesthetic for MRI, but results were inconclusive given that the patient was under sedation. The constellation of signs and symptoms such as cleft palate at birth, progressive joint contractures of the feet and knees (pterygium like), vertebral and rib abnormalities, decreased range of motion due to myotonia, congenital vertical talus deformity of the left foot, abnormally low body weight, and mask‐like facies raised enough concerns to treat her with similar anesthesia precautions due to her presumptive diagnosis. Given the unfavorable airway features, prudence is required when preparing for airway instrumentation as described in this case. Despite the history of previous straightforward intubations, this patient was older and likely showing signs of disease progression which necessitated the use of video laryngoscopy for airway instrumentation.

Malignant hyperthermia susceptibility in SJS has also been discussed in the literature, but the relationship remains uncertain, with some reports suggesting an association, while others dispute a direct link [13–15]. In our case, malignant hyperthermia was not treated as a primary anticipated complication based on the patient’s anesthetic history, which included multiple prior general anesthetics without hypermetabolic events. Nonetheless, given the broader concern for perioperative complications in rare neuromuscular conditions, we maintained routine intraoperative vigilance of hypercarbia, hyperthermia, rigidity, and metabolic acidosis, with readiness to escalate management if concerning signs emerged.

The most unusual aspect of this case was the presence of significant pulmonary adhesions during thoracoscopic surgery in a patient with no known risk factors. Typical causes of pleural adhesions include prior chest trauma, previous thoracic surgery, pleural or pulmonary infections like tuberculosis, and malignancy. Given such, the presence of significant adhesions is of importance to the anesthesiologist as it will require the surgeon to perform adhesiolysis, prolong the operative time, increase bleeding, and increase the risk of damage to lung parenchyma. In this case, thoracoscopic surgery was still technically feasible, but consideration for conversion to an open procedure should be considered in the development of an anesthetic plan.

Following the thoracoscopic surgery, the posterior spine surgery was complicated by ventilation difficulties and subsequent discovery of a sanguinous mucus plug. This potentially suggests that blood and inflammatory debris from the unexpected challenges during the first stage of surgery contributed to the development of a nearly occlusive endotracheal tube plug. Lung isolation was not obtained; instead, tidal volumes were lowered, and CO_2_ gas insufflation was used to aid visualization. Lung isolation with a double lumen tube or bronchial blocker could have possibly decreased airway contamination though not necessarily prevent endotracheal tube obstruction. The patient also demonstrated reduced glottic‐to‐carina distance (tracheal shortening) that predisposed to mainstem intubation during the subsequent completion PSF, adding to the anesthetic complexity.

The perioperative course of this spinal fusion patient with suspected SJS is the first report to our knowledge in the literature. Preoperative evaluation for pleural adhesions is a consideration for perioperative optimization, as lung ultrasound and respiratory dynamic computed tomography are clinically useful to detect pleural adhesions [16, 17]. However, this patient did not have any signs or symptoms that would suggest a need for such investigation, and no association of pleural adhesions in patients with SJS has been established. Additionally, when a complex patient is undergoing a combined anterior spinal fusion and PSF and unexpected pulmonary adhesions are encountered on the anterior portion, a deliberate surgical pause halfway between procedures could be considered. At the conclusion of the anterior portion of the case, the patient should be repositioned supine. The endotracheal tube should be lavaged and suctioned; a chest X‐ray should be performed to confirm appropriate endotracheal tube placement, prior to positioning prone. This could allow for reassessment of pulmonary gas exchange, airway pressures, patency of the endotracheal tube, bronchoscopic evaluation of the airway, and discussion of the likely disposition of the patient should the second half of the procedure be continued.

The principal message from this case, however, is not that endotracheal tube obstruction or malpositioning is unique to SJS. Rather, these issues are poorly tolerated in a syndromic patient with severe restrictive lung disease, distorted tracheal anatomy, and limited physiologic reserve undergoing a surgery with lung manipulation, complex adhesiolysis, and surgical position changes. The threshold to reevaluate endotracheal tube patency and position in similar cases should be low. In this case, anticipation of a difficult airway, prompt bronchoscopy when a suction catheter could not be passed in the endotracheal tube, safe repositioning from a prone to supine position, and accommodation of a foreshortened trachea contributed to an ultimately successful outcome.

## 4. Conclusion

This case demonstrates the successful perioperative management of a patient with presumed SJS undergoing complex anterior spinal release and PSF. Unexpected dense pleural adhesions increased the surgical complexity, but an association for such with SJS remains uncertain. Following the anterior release and repositioning for posterior fusion, the patient developed significant intraoperative endotracheal tube obstruction from a sanguineous mucus plug. Prompt recognition, bronchoscopic evaluation, supine repositioning, and clearance of the obstruction restored ventilation, and the posterior fusion was postponed. The patient returned 1 week later for completion of the posterior fusion, during which challenges with endotracheal tube placement depth due to a foreshortened trachea were managed, allowing the procedure to be completed safely. Although endotracheal tube obstruction and tube malpositioning are not unique to SJS, heightened vigilance is particularly important during prolonged anterior and posterior spinal procedures involving thoracic manipulation and positional changes.

## Funding

No funding was received for this manuscript.

## Conflicts of Interest

Amy McIntosh is a consultant for Medtronic and Globus. The other authors declare no conflicts of interest.

## Supporting Information

Additional supporting information can be found online in the Supporting Information section.

## Supporting information


**Supporting Information** CARE‐checklist v2.

## Data Availability

The data that support the findings of this study are available from the corresponding author upon reasonable request.
